# Occupational characteristics associated with SARS-CoV-2 infection in the UK Biobank during August–November 2020: a cohort study

**DOI:** 10.1186/s12889-022-14311-5

**Published:** 2022-10-10

**Authors:** Elizabeth L. Yanik, Bradley A. Evanoff, Ann Marie Dale, Yinjiao Ma, Karen E. Walker-Bone

**Affiliations:** 1 Department of Orthopaedic Surgery, Washington University School of Medicine, 660 S. Euclid Ave, Campus Box 8233, St. Louis, MO 63110 USA; 2 Department of Surgery, Washington University School of Medicine, St. Louis, MO USA; 3 Division of General Medical Sciences, Washington University School of Medicine, St. Louis, MO USA; 4 Division of Biostatistics, Washington University School of Medicine, St. Louis, MO USA; 5 Monash Centre for Occupational and Environmental Health, Monash University, Melbourne, Victoria Australia

**Keywords:** COVID-19, Occupational health, Job exposure matrix, UK Biobank

## Abstract

**Background:**

Occupational exposures may play a key role in severe acute respiratory syndrome coronavirus 2 (SARS-CoV-2) infection risk. We used a job-exposure matrix linked to the UK Biobank to measure occupational characteristics and estimate associations with a positive SARS-CoV-2 test.

**Methods:**

People reporting job titles at their baseline interview in England who were < 65 years of age in 2020 were included. Healthcare workers were excluded because of differential access to testing. Jobs were linked to the US Occupational Information Network (O*NET) job exposure matrix. O*NET-based scores were examined for occupational physical proximity, exposure to diseases/infection, working outdoors exposed to weather, and working outdoors under cover (score range = 1–5). Jobs were classified as remote work using two algorithms. SARS-CoV-2 test results were evaluated between August 5th-November 10th, 2020, when the UK was released from lockdown. Cox regression was used to calculate adjusted hazard ratios (aHRs), accounting for age, sex, race, education, neighborhood deprivation, assessment center, household size, and income.

**Results:**

We included 115,451 people with job titles, of whom 1746 tested positive for SARS-CoV-2. A one-point increase in physical proximity score was associated with 1.14 times higher risk of SARS-CoV-2 (95%CI = 1.05–1.24). A one-point increase in the exposure to diseases/infections score was associated with 1.09 times higher risk of SARS-CoV-2 (95%CI = 1.02–1.16). People reporting jobs that could not be done remotely had higher risk of SARS-CoV-2 regardless of the classification algorithm used (aHRs = 1.17 and 1.20). Outdoors work showed an association with SARS-CoV-2 (exposed to weather aHR = 1.06, 95%CI = 1.01–1.11; under cover aHR = 1.08, 95%CI = 1.00–1.17), but these associations were not significant after accounting for whether work could be done remotely.

**Conclusion:**

People in occupations that were not amenable to remote work, required closer physical proximity, and required more general exposure to diseases/infection had higher risk of a positive SARS-CoV-2 test. These findings provide additional evidence that coronavirus disease 2019 (COVID-19) is an occupational disease, even outside of the healthcare setting, and indicate that strategies for mitigating transmission in in-person work settings will remain important.

## Background

Many severe acute respiratory syndrome coronavirus 2 (SARS-CoV-2) outbreaks have been documented in workplace settings [1, 2], and for those doing in-person work, the workplace can be a key point of exposure to SARS-CoV-2 [3]. Most studies of workplace risk for SARS-CoV-2 infection have focused on specific occupational groups with particularly high risk, such as healthcare workers [4–7]. A smaller proportion of studies have examined a general population sample to investigate occupational characteristics associated with SARS-CoV-2 infection across a diverse array of job types [8, 9]. The UK Biobank, a 500,000 person cohort with linked SARS-CoV-2 testing data, provides an ideal resource for such a study.

One of the first studies of occupational risk for SARS-CoV-2 in the UK Biobank demonstrated that early in the pandemic, essential workers had increased risk of developing severe coronavirus disease 2019 (COVID-19), particularly those in the healthcare and social/education sectors [10]. Other studies have demonstrated that shift work is associated with higher risk of severe COVID-19 [11, 12]. However, these studies had a limited number of occupational characteristics that could be examined, and were conducted whilst the UK was in lockdown (essential work only).

We aimed to leverage a job exposure matrix that was recently linked to job titles collected in the UK Biobank [13] in order to examine associations between several occupational characteristics and SARS-CoV-2 exposure risk. Our study focused on a time period after the UK was released from lockdown when workers in a greater variety of jobs would have been expected to return to in-person work.

## Methods

### Study population

Between 2006 and 2010, the UK Biobank enrolled approximately 500,000 people aged 40–69 years and registered with the National Health Service [14, 15]. At their baseline assessment visit, participants gave informed consent and completed detailed questionnaires providing information on demographics, health behaviors, and other characteristics. At the same visit, a verbal interview was conducted by trained UK Biobank staff which included recording the participant’s current job title which was automatically coded according to the UK Standard Occupational Classification (SOC) System 2000 [16] for all participants who were employed or self-employed [17]. Our research team previously linked UK Biobank job titles to a job exposure matrix based on the U.S. Occupational Information Network (O*NET) database and evaluated the validity of O*NET scores in the UK Biobank population [13]. After the start of the COVID-19 pandemic, the UK Biobank cohort was linked to Public Health England’s Second Generation Surveillance System to obtain SARS-CoV-2 nucleic acid detection test results starting in March 2020 [18].

The current study was limited to participants reporting a job title at their baseline assessment visit in the UK Biobank that could be linked to the O*NET job exposure matrix (JEM) [13] (178,518 participants did not report a current job title at baseline, while 895 participants had job titles that did not link to complete information on Work Context scores in O*NET). We excluded people 65 years of age or older by 2020, as these individuals were more likely to be retired and no longer working in the job they reported at their baseline assessment visit. Healthcare workers were excluded from these analyses because of their differential access to testing. Specifically, people in many healthcare occupations were tested weekly regardless of symptoms during the time period of our study [19, 20]. Additionally, participants recruited from assessment centers in Scotland and Wales were excluded as UK Biobank had only linked SARS-CoV2 testing from England at the time of our analysis (close to 90% of the UK Biobank cohort comes from England).

### Characterization of occupations

The O*NET JEM characterizes jobs on numerous dimensions [21]. For this study, we selected four O*NET variables of interest that characterize relevant aspects of physical work conditions, each with values ranging from one to five. This included ‘Physical Proximity’, a variable that captured distance from other workers, with a value of five indicating workers were near touching and a value of one indicating workers are beyond 100 ft. apart. Additionally, three variables collected time-based exposures on how frequently a job required work that was ‘Exposed to Disease or Infections’, ‘Outdoors, Exposed to Weather’ and ‘Outdoors, Under Cover’, respectively, with a value of five indicating exposure every day and a value of one indicating that exposure was never required. Scores for all O*NET variables are based on job analysts’ ratings and worker questionnaires that were administered before the COVID-19 pandemic. Therefore, SARS-CoV-2 exposures were not considered as part of the assessment of the ‘Exposed to Disease or Infections’ measure. Job titles reported by UK Biobank participants were mapped to US SOC 2010 codes allowing assignment of O*NET scores for each variable to UK Biobank participants [13]. Some UK Biobank job titles mapped to multiple US SOC 2010 codes. For participants in jobs mapped to multiple US SOC codes, the average of the O*NET scores was used.

We also constructed two measures capturing whether or not remote work was possible using O*NET variables. The first measure uses seven O*NET variables characterizing work context and eight O*NET variables characterizing work activities to classify remote work as described by Dingel & Neiman [22] (hereafter referred to as the Dingel Index). Some UK Biobank participants that mapped to multiple US SOC 2010 codes could be classified as either remote or non-remote work depending on the code. We classified these individuals into a ‘maybe’ remote work category. The second measure, developed by Baker, classifies remote work jobs based on two O*NET items related to remote activities (“Importance of Computer Use”) and public activities (“Importance of Interaction with the Public”) [23] (hereafter referred to as the Baker Index). People were categorized as remote work if their job had both an “Importance of Computer Use” score greater than three and an “Importance of Interaction with the Public” score of less three, while all others were classified as non-remote work.

### Outcome definition

Our primary outcome of interest was the first positive SARS-CoV-2 test result occurring between August 6th and November 10th, 2020. The UK was not under lockdown and most businesses were allowed to open by Aug. 1st, 2020 (Fig. 1, [24, 25]). A second lockdown was declared on Nov. 5th, 2020. Our time interval was chosen to correspond with the window of time when lockdown measures were not in effect, with a five-day lag added to account for the median incubation period between initial SARS-CoV-2 exposure/infection and development of symptoms [26]. Notably, this time period also occurs before the availability of COVID-19 vaccines, and so results were not influenced by differential vaccine uptake across occupations.

**Figure:**
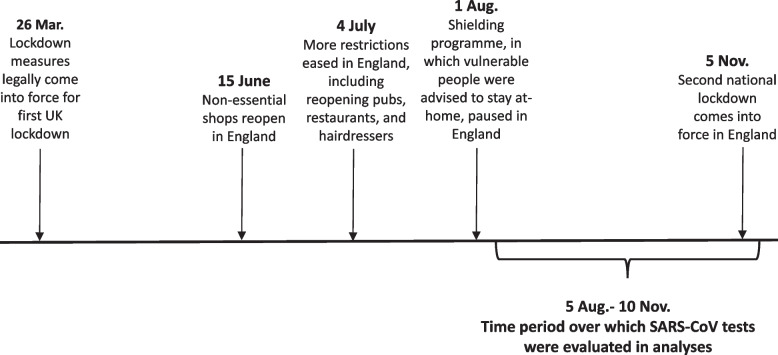
**Fig. 1** Timeline of lockdown measures in England during 2020 [24, 25]

### Statistical analyses

Distributions of the occupational characteristics of interest were described across the total study population and among participants with a positive SARS-CoV-2 test between Aug. 6th and Nov. 10th, 2020. Cox regression was used to calculate hazard ratios as estimates of association between each occupational characteristic and time to a first positive SARS-CoV-2 test. Multivariable Cox regression was used to calculate adjusted hazard ratios (aHRs), accounting for age, sex, race, Townsend Deprivation Index (measure of neighborhood socioeconomic status), education, UK Biobank assessment center, household size, and income. Sensitivity analyses were undertaken to consider results with and without adjustment for race.

As high scores for ‘Outdoors, Exposed to Weather’ and ‘Outdoors, Under Cover’ may in part reflect an inability to work from home, associations were estimated for these variables after additionally adjusting for remote work. For these O*NET variables, two Cox regression models were run that adjusted for each of the two remote work indices in turn (Dingel index and Baker index), as well as the variables in the initial multivariable analyses.

The National Health Service Research Ethics Committee approved UK Biobank. The Washington University Institutional Review Board determined this study to be exempt from oversight.

## Results

**Fig. 2:**
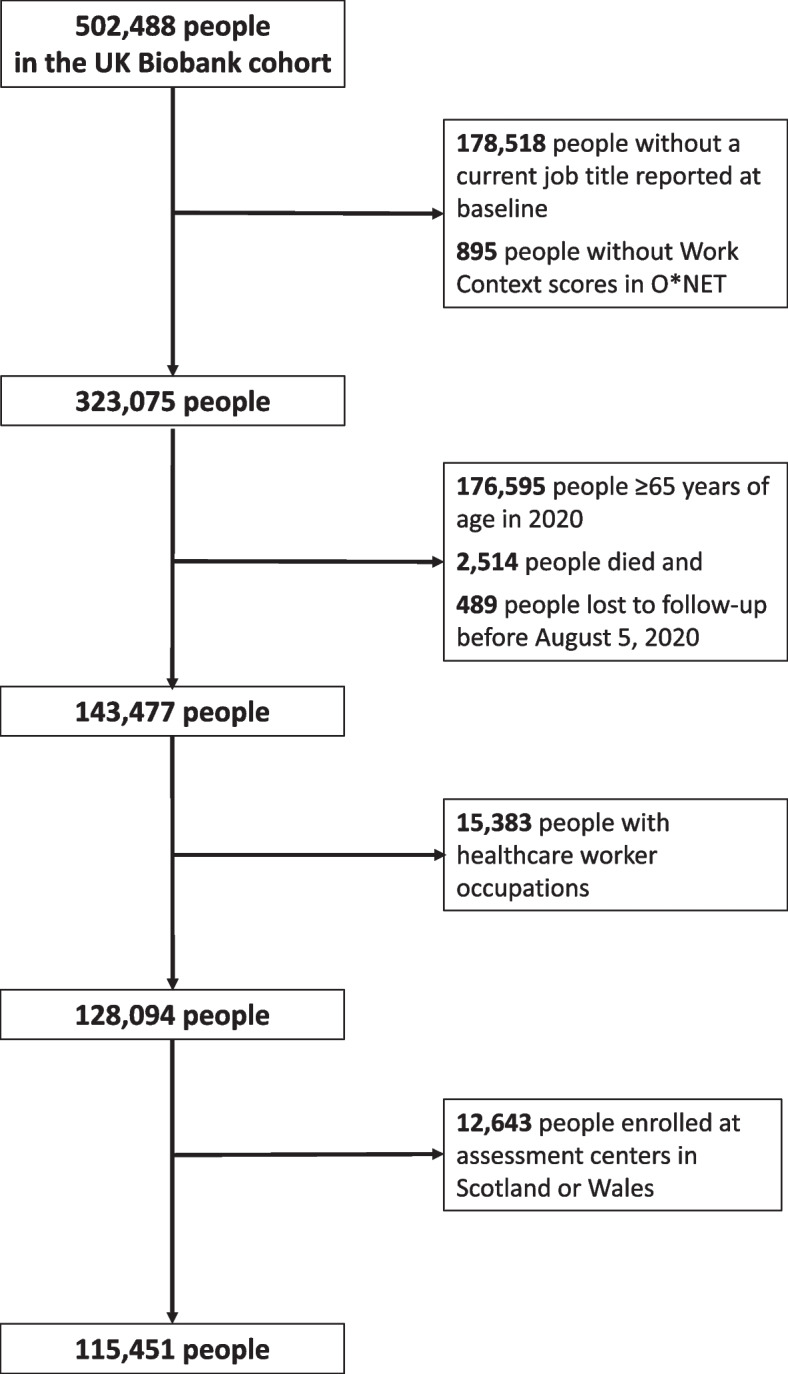
Selection of UK Biobank study population

The UK Biobank cohort included 502,488 participants at the time of this study, 323,075 of whom reported a job at their baseline assessment visit that could be linked to O*NET information (Fig. 2). Of those, 143,477 (44.4%) were less than 65 years of age and still in follow-up in the UK Biobank by August 2020. Finally, 15,383 health care workers and 12,643 participants enrolled in Scotland and Wales were excluded. The remaining 115,451 people made up the final analytic population (Fig. 2). In this population, 47.8% were male, 91.3% were White, and 38.6% had college or University education (Table 1). People living alone made up 14.3% of the population, while 0.21% had eight or more people in their household.

**Table 1:** Characteristics of 115,451 people less than 65 years of age in 2020 who reported job titles at UK Biobank assessment centers in England

Characteristic	N (%)/Median (IQR)
Approximate Age by March 2020 in years, Median (IQR)	58 (55,62)
Male Sex, N (%)	55,223 (47.83)
Race, N (%)^a^	
White	105,373 (91.27)
Black	3194(2.77)
Asian	3800(3.29)
Mixed	1203 (1.04)
Unknown	1881 (1.63)
Townsend deprivation index, Median (IQR)^a^	−1.9(−3.5,0.8)
Highest Level of Education, N(%)	
College/University	44,535 (38.57)
Other professional qualifications (e.g. nursing, teaching)	13,670 (11.84)
NVQ, HND, HNC, or equivalent	19,620 (16.99)
A levels/AS levels or equivalent	7848 (6.80)
O levels/GCSEs/CSEs or equivalent	22,464 (19.46)
None of the above	5648 (4.89)
Unknown	1666 (1.44)
Number in household, N(%)^a^	
1	16,534 (14.32)
2	28,560 (24.74)
3	23,746 (20.57)
4	33,231 (28.78)
5+	12,773 (11.06)
Unknown	604 (0.52)
Average total household income before tax, N(%)^a^	
< 18,000	9766 (8.46)
18,000 to 30,999	19,895 (17.23)
31,000 to 51,999	33,493 (29.01)
52,000 to 100,000	33,052 (28.63)
> 100,000	8638 (7.48)
Unknown	10,607 (9.19)
Major Occupational Groups, N(%)^b^	
Managers and Senior Officials	23,697 (20.53)
Professional Occupations	24,908 (21.57)
Associate Professional and Technical Occupations	17,860 (15.47)
Administrative and Secretarial Occupations	17,997 (15.59)
Skilled Trades Occupations	8811 (7.63)
Personal Service Occupations	7254 (6.28)
Sales and Customer Service Occupations	3919 (3.39)
Process, Plant and Machine Operatives	5069 (4.39)
Elementary Occupations	5936 (5.14)

^a^Race unknown for 1881 people. Townsend Deprivation Index missing for 187 people. Number in household missing for 604 people. Average total household income before tax missing for 10,607 people

^b^Major group classifications based on UK Standard Occupational Classification 2000

*IQR* Interquartile range

The distribution of O*NET job characteristics was assessed in the study population. For ‘Exposed to Disease or Infections’, ‘Outdoors, Exposed to Weather’ and ‘Outdoors, Under Cover’ most people had jobs that required less than monthly exposure (O*NET scores < 3) (Table 2). For ‘Physical Proximity’, less than 25% of people had jobs that required working within arm’s length of other people (O*NET score > =4). For all O*NET variables, scores were higher for people with a positive SARS-CoV-2 test, indicating more exposure to diseases/infection, more outdoors work, and closer physical proximity to other people during work (Table 2). Using the Dingel remote work index, 53.9% of the population was identified as having jobs that could be done remotely, while 45.7% of people testing positive for SARS-CoV-2 were identified as having jobs that could be done remotely. Using the Baker remote work index, 23.8% of the population was identified as having jobs that could be done remotely, while 19.2% of people testing positive for SARS-CoV-2 were identified as having jobs that could be done remotely (Table 2). Seventy-four percent of people identified as doing remote work by the Baker index were also identified as doing remote work by the Dingel index (not including those identified as ‘maybe’ doing remote work). However, as the Dingel index identified many more remote work jobs, only 33% of these matched with the Baker index definition of remote work.

**Table 2:** Distributions of Occupational Characteristics derived from O*NET

Occupational Characteristics	N (%)/Median (IQR)	
	Total Population	People with a positive SARS-CoV-2 test
**Total N**	**115,451**	**1746**
Exposed to Disease or Infections Score, Median (IQR)	1.36(1.17, 2.11)	1.41 (1.18, 2.18)
Outdoors, Exposed to Weather Score, Median (IQR)	1.73(1.29, 2.77)	1.96 (1.34, 2.92)
Outdoors, Under Cover Score, Median (IQR)	1.41(1.15, 1.93)	1.47 (1.15, 2.05)
Physical Proximity Score, Median (IQR)	3.13(2.83, 3.78)	3.21 (2.89, 3.87)
Work can be done remotely [Dingel index]^a^, n(%)		
Yes	62,293(53.90)	797 (45.65)
Maybe	7839(6.78)	110 (6.30)
No	45,449(39.32)	839 (48.05)
Work can be done remotely [Baker index]^a^, n(%)		
Yes	27,531(23.8)	335 (19.19)
No	88,050(76.2)	1411 (80.81)

^a^20,249 people had jobs that were identified as ‘Yes’ remote work by the Dingel index and ‘Yes’ remote work by the Baker index, including 230 people with a SARS-CoV-2 test

*IQR* Interquartile range, O*NET Occupational information network

In unadjusted analyses, more frequent occupational exposure to diseases/infections, more frequent exposure to outdoors work exposed to weather, more frequent exposure to outdoors work under cover, and working in closer proximity to other people were each associated with a higher risk of a positive SARS-CoV-2 test (Table 3). Having a job that could not be done remotely was also associated with a higher risk of a positive SARS-CoV-2 test, regardless of the classification system used to define remote work. After adjusting for age, sex, race, Townsend Deprivation Index, education, UK Biobank assessment center, household size, and income, all associations between occupational exposures and risk of a positive SARS-CoV-2 test were attenuated, but remained statistically significant (Table 3). For individual O*NET variables, the strongest association was observed for physical proximity with each 1-point increase associated with a 14% increased risk of a positive SARS-CoV-2 test (95%CI: 5, 24%). This was followed by exposure to disease/infections, for which a 1-point increase was associated with 9% increased risk of a positive SARS-CoV-2 test (95%CI: 2, 16%). Having a job that could not be done remotely was associated with 17–20% increased risk of a positive SARS-CoV-2 test, depending upon the classification system (Dingel index aHR = 1.17, 95%CI = 1.05–1.31; Baker index aHR = 1.20, 95%CI = 1.06–1.36; Table 3). Associations were the same with and without adjustment for race (Table 4).

**Table 3:** Associations with a positive SARS-CoV-2 test between Aug. 5, 2020 and Nov. 10, 2020

Occupational Characteristics	Unadjusted HR (95%CI)	Adjusted HR^a^ (95%CI)
Exposed to Disease or Infections Score	1.11 (1.05–1.18)	1.09 (1.02–1.16)
Outdoors, Exposed to Weather Score	1.13 (1.08–1.18)	1.06 (1.01–1.11)
Outdoors, Under Cover Score	1.19 (1.11–1.27)	1.08 (1.00–1.17)
Physical Proximity Score	1.20 (1.11–1.29)	1.14 (1.05–1.24)
Work can be done remotely [Dingel index]		
Yes	Reference	Reference
Maybe	1.10 (0.90–1.34)	1.10 (0.89–1.35)
No	1.45 (1.31–1.60)	1.17 (1.05–1.31)
Work can be done remotely [Baker index]		
Yes	Reference	Reference
No	1.32 (1.17–1.49)	1.20 (1.06–1.36)

^a^Cox regression adjusted for age, sex, race, Townsend Deprivation Index, education, assessment center, number in household, and income

*HR* Hazard ratio, *CI* Confidence interval

**Table 4:** Associations with a positive SARS-CoV-2 test between Aug. 5, 2020 and Nov. 10, 2020 without adjustment for race

Occupational Characteristics	Adjusted HR^a^ (95%CI)
Exposed to Disease or Infections Score	1.09 (1.02–1.16)
Outdoors, Exposed to Weather Score	1.05 (1.00–1.11)
Outdoors, Under Cover Score	1.08 (1.00–1.17)
Physical Proximity Score	1.14 (1.05–1.24)
Work can be done remotely [Dingel index]	
Yes	Reference
Maybe	1.10 (0.89–1.35)
No	1.17 (1.04–1.31)
Work can be done remotely [Baker index]	
Yes	Reference
No	1.20 (1.06–1.36)

^a^Cox regression adjusted for age, sex, Townsend Deprivation Index, education, assessment center, number in household, and income

*HR* Hazard ratio, *CI* Confidence interval

For both outdoors work under cover and outdoors work exposed to weather, associations with a positive SARS-CoV-2 test were attenuated and no longer statistically significant after adjusting for remote work (Adjusted for Dingel Index: ‘Outdoors, Exposed to Weather’ aHR = 1.03 [95%CI = 0.98–1.09], ‘Outdoors, Under Cover’ aHR = 1.04 [95%CI = 0.95–1.13]; Adjusted for Baker Index: ‘Outdoors, Exposed to Weather’ aHR = 1.04 [95%CI = 0.99–1.10], ‘Outdoors, Under Cover’ aHR = 1.06 [95%CI = 0.98–1.15]).

## Discussion

To understand relationships between occupational characteristics and SARS-CoV-2 risk, our study capitalized on a large population-based cohort with a wide variety of occupations during a particularly important time period in the UK when most businesses were open, but vaccines were not yet available. In this population we identified associations between several different occupational characteristics and the risk of a positive SARS-CoV-2 test, indicating that in-person work is an important contributor to SARS-CoV-2 transmission.

Several studies have examined associations between work and risk of severe COVID-19 in the UK Biobank. Early in the pandemic, Mutambudzi et al. found that essential workers, particularly healthcare workers and social/education workers, had higher risk of severe COVID-19 than non-essential workers [10]. Subsequent studies identified similar findings, as well as an association between shift work and higher risk of severe COVID-19 [11, 12]. In contrast to these studies, our study focused on the time period after the UK emerged from lockdown, included occupational characteristics that were not previously examined, and used SARS-CoV-2 positive tests as our primary outcome. However, consistent with these prior studies, our results indicate that in-person work is associated with higher risk of SARS-CoV-2 infection. Importantly, our study demonstrates that this elevated risk is not limited to healthcare workers as this occupational group was excluded in our analysis.

Several of the occupational risk factors we identified were consistent with our initial hypotheses and with studies in other populations. Closer physical proximity and higher exposure to non-SARS-CoV-2 infections and diseases were associated with higher risk of SARS-CoV-2 infection. A prior UK Biobank study by Maidstone et al. identified a positive correlation between SARS-CoV-2 positive tests and a higher work environment score that combined measures for infection exposure and proximity [11]. But the study did not account for potential confounders of this relationship, such as household size or measures of socioeconomic status [11]. A national study in the UK also demonstrated that COVID-19 death rates in 2020 were higher among people working in industries that would typically require more exposure to infections and close proximity to others (ex. taxi drivers and care workers) [27]. Our study also found that having a job that was not amenable to remote work increased risk of a SARS-CoV-2 infection. This is consistent with case-control studies in several other countries, in which telework has been associated with decreased SARS-CoV-2 infection risk [28–30].

We hypothesized that working outdoors would decrease SARS-CoV-2 exposure, but found that outdoors work was associated with higher rates of SARS-CoV-2 infection. This association was attenuated after adjusting for remote work, indicating that our initial association estimates were partly attributable to the fact that outdoors work cannot typically be done remotely. However, there was still no indication that outdoors work reduced SARS-CoV-2 infection risk. Even jobs that typically require significant amounts of time spent outside, may also include time spent in high-risk indoor settings potentially negating any beneficial effect of time spent outdoors. For example, investigations of COVID-19 outbreaks occurring at construction sites after resumption of non-essential work in the United States revealed that lunches and breaks were frequently taken in indoors areas with poor ventilation [31]. Similarly, police officers have some of the highest O*NET scores for time spent outside, but their job duties may also put them in situations with high-exposure to SARS-CoV-2. An analysis of the UK Office of National Statistics COVID-19 Infection Survey found police and protective services workers to have particularly high risk of SARS-CoV-2 infection compared to other types of workers [32].

Our study had a number of strengths. UK Biobank represents a large sample of the general population in the UK that includes a wide diversity of occupations. We evaluated new SARS-CoV-2 infections during a critical time period in which the economy was open, but vaccines were not yet available to provide protection. Through use of a linked JEM we were able to evaluate a number of job characteristics potentially relevant to SARS-CoV-2 exposure [13]. Finally, we were able to control for a number of relevant sociodemographic variables that could influence SARS-CoV-2 infection risk, such as household size.

Our study also has several limitations that should be acknowledged. First, occupational characteristics were assigned based on jobs that were reported at the baseline assessment visit in the UK Biobank, which may not represent the work participants were doing during the study period. Participants may have changed jobs in the intervening years, or may have been out-of-work due to the pandemic. The bias arising from this misclassification would be expected to bias estimates towards the null hypothesis (i.e. no association) and could lead to underestimation of true associations. Second, we could not account for differences in SARS-CoV-2 mitigation strategies between workplaces. Settings in which workers have more physical proximity or known exposure to people with infections may have more mitigation measures in place. For example, in the healthcare setting, SARS-CoV-2 prevention measures are typically more stringent and healthcare workers were routinely tested for SARS-CoV-2 in the UK during the time period of this study. Our study excluded healthcare workers due to the known differences in COVID-19 testing, but there were likely differences in mitigation measures across the other occupations included in our study. Finally, while our use of a JEM eliminates the recall bias that can be inherent in self-reported job exposures, a JEM only allows us to capture average exposures at the job-level, without capturing worker-level variability. As a result, this introduces non-differential measurement error in our assessment of occupational exposures.

## Conclusions

In conclusion, we found that people in occupations that are less conducive to remote work have higher risk of a positive SARS-CoV-2 test. Higher workplace physical proximity to other people and higher general exposure to infections and disease were associated with increased risk of a positive SARS-CoV-2 test. People doing jobs requiring outdoors work did not have lower risk of SARS-CoV-2, highlighting the potential risks of any kind of in-person work. In sum, these findings provide additional evidence that COVID-19 is an occupational disease, even outside of the healthcare setting. While vaccines are now widely available to limit the impact of SARS-CoV-2, waves of infections continue and strategies for mitigating transmission in all in-person work settings will remain important.

## Data Availability

This research has been conducted using the UK Biobank resource under application 27034.The datasets supporting the conclusions of this article are available in the UK Biobank data repository, [https://www.ukbiobank.ac.uk/]. All bona fide researchers can apply to access the UK Biobank data repository to conduct health-related research that is in the public interest.

## Abbreviations

SARS-CoV-2: Severe acute respiratory syndrome coronavirus 2
COVID-19: Coronavirus disease 2019
SOC: Standard Occupational Classification
O*NET: Occupational Information Network
JEM: Job Exposure Matrix
IQR: Interquartile range
aHR: adjusted Hazard Ratio
CI: Confidence interval
